# Influence of Three Probiotics Strains, *Lactobacillus rhamnosus* GG, *Bifidobacterium animalis* subsp. *Lactis* BB-12 and *Saccharomyces boulardii* CNCM I-745 on the Biochemical and Haematological Profiles and Body Weight of Healthy Rabbits

**DOI:** 10.3390/biology10111194

**Published:** 2021-11-17

**Authors:** Louiza Kadja, Amira Leila Dib, Nedjoua Lakhdara, Assia Bouaziz, Elena Espigares, Mohammed Gagaoua

**Affiliations:** 1 Gestion Santé et Productions Animales Research Laboratory, Institut des Sciences Vétérinaires El-Khroub, Université Frères Mentouri Constantine 1, Constantine 25000, Algeria; louiza20132014@gmail.com (L.K.); dib.amiraleila@umc.edu.dz (A.L.D.); lakhdara.nedjoua@umc.edu.dz (N.L.); bouaziz.assialp@gmail.com (A.B.); 2 Department of Preventive Medicine and Public Health, Faculty of pharmacy, University of Granada, 18071 Granada, Spain; elespi@ugr.es; 3 Food Quality and Sensory Science Department, Teagasc Food Research Centre, Ashtown, D15 KN3K Dublin, Ireland

**Keywords:** rabbits, diet, probiotics, immunity, health, microbiota, body weight

## Abstract

**Simple Summary:**

Currently, probiotics are used as growth promoters on a large scale to improve the productivity of several animals’ species within the aim of reducing the presence of antibiotic residues in animal products consumed by humans. Several reports evidenced the positive effect of probiotic supplementation on the growth performances and health of rabbits, mainly through the balance of the intestinal microbiota of the host animal. Therefore, certain probiotics, including *Lactobacilli*, *Bifidobacteria*, *Saccharomyces*, can improve the biochemical and haematological profiles, especially in production animals. In this context, this study was performed on rabbits for the economic importance they play as a source of meat proteins in developing countries and their use as experimental models in research and biomedicine. This study then aimed to evaluate the effect of three strains of probiotics: *Lactobacillus rhamnosus* GG *Bifidobacterium animalis* subsp. *Lactis* BB-12 and *Saccharomyces boulardii* CNCM I-745, on the biochemical and haematological parameters and their influence on the rabbit’s weight of the ITELV2006 strain. The findings evidenced that the probiotic strain affected the biochemical and haematological parameters. Further, the strains showed a positive effect on the weight gain of the rabbits.

**Abstract:**

This study aimed to investigate the effects of three strains of probiotics, these being *Lactobacillus rhamnosus* GG, *Bifidobacterium animalis* subsp. *Lactis* BB-12 and *Saccharomyces boulardii* CNCM I-745, on the body weight, animal performances and blood parameters of rabbits (male and female) of the ITELV2006 strain. The supplementation of the feed of the rabbits with the three probiotic strains allowed observing positive effects on most of the biochemical and haematological parameters investigated during a period of 60 days (30 days of supplementation and 30 days without treatment). Further, there was a significant improvement in the body weight of the rabbits at the end of the experiment. The effect of the three probiotics investigated in this trial was found to be related to the sex of the rabbits and to the intake period (duration). Ultimately, these findings raise the possibility of using probiotics to investigate in an in-depth and specific manner based on fixed factors such as the strain, the gender and age of the animals, the main underlying mechanisms and effects, which would allow achieving optimal and adapted health benefits and sustainable production. In the context of animal production, it is worth investigating in a targeted study the effect of the three strains on muscle growth and development and finding evidence of the possible consequences on meat quality traits of the rabbits supplemented with probiotics.

## 1. Introduction

The growth of the world population is related to an increased demand for food of plant and animal origins. Thus, it is estimated that by 2050, the world population will reach around 9 billion, where the majority of this augmentation will take place in developing countries [1]. Therefore, the food security concern will become alarming. Further, developing countries are often characterised by a deficiency in animal proteins. Subsequently, to reduce the deficiency in animal protein supply to populations, it is a prerequisite to encourage the breeding of animals with a short life cycle such as rabbits [2]. Indeed, rabbit meat is characterised by its high nutritional and dietary qualities and had one of the highest percentages in protein content [3], low fat [4] and a lower caloric value (160 Kcal/100 g of meat); therefore, it can be consumed by heart patients and children. Furthermore, rabbit is a herbivore capable of making good use of several plant sources and by-products of the agri-food industries without any competition to those of humans [5].

However, the efficiency and economy of rabbit production are largely threatened by the appearance of digestive diseases mainly after weaning [6]. In fact, the intestinal microbiota seems to have a major importance in maintaining digestive health [7]. Several studies highlighted the interactions between the host and the gut microbiota and proved its vital role [8]. Actually, it is well-known that the microbiota is involved in metabolic, trophic and protective functions and can convert a wide variety of substrates (carbohydrates, proteins and lipids), hence generating plenty of metabolites with beneficial effects [9]. Furthermore, the microbiota has a role in regulating the renewal of cells in the intestinal wall and provides protective functions by limiting the implantation of pathogenic bacteria through the mechanisms of resistance to colonisation and by stimulating the immune system [10]. Thus, due to its numerous essential physiological functions, the idea of positively modulating the microbiota to meet the production objectives is explored by the administration of living microorganisms known as probiotics [11]. According to experts from the Food and Agriculture Organisation (FAO) and World Health Organisation (WHO) working groups, probiotics are defined as live strains of strictly selected microorganisms that confer a health benefit on the host when administered in sufficient quantities [12]. Nowadays, they are the subject of numerous studies regarding their effectiveness in improving the zootechnical characteristics of livestock (feed conversion rate and daily weight gain) [13], or for the prevention of various pathologies [14].

The objective of this work is to study the effect of three different probiotics (*Lactobacillus rhamnosus* GG, *Bifidobacterium animalis* subsp. *Lactis* BB-12 and *Saccharomyces boulardii* CNCM I-745) during and after their administration (30 days of supplementation and 30 days without treatment), on weight, biochemical and haematological parameters of rabbits of the ITELV2006 strain. To the best of the authors’ knowledge, this study is among the first attempts to investigate the influence of these three probiotic strains on the metabolism of rabbits and ITELV2006 strain.

## 2. Materials and Methods

### 2.1. Animals, Breeding Conditions and Experimental Design

Forty rabbits of the ITELV2006 strain of both sexes (ratio 50:50, females and males), weaned and aged 5 weeks (599.16 ± 27.01 g) were used in this trial. The strain is a genotype obtained by crossing between males of selected paternal strain, INRA2666 (France) and local white female populations [15,16]. All the animals were subjected to the same breeding conditions and were housed in individual cages (0.6 × 0.5 × 0.25 m^3^) mounted in a battery. A cycle of 16 h light/8 h dark was maintained throughout the experiment. The temperature and humidity were recorded continuously and are in the range of 20 ± 1.5 °C and 60 ± 5%, respectively. All the rabbits were fed ad libitum with commercial pellets (staple feed) and had free access to water.

For the experiment, the rabbits were randomly divided into four groups of 10 animals; each group consisted of 5 males and 5 females. The experiment was undertaken over 60 days (30 days of supplementation and 30 days without treatment). Briefly, the animals received the probiotics diluted in 1 mL of sterile water by means of oral gavage daily during a period of one month following an earlier procedure [17]. The three probiotics, *Lactobacillus rhamnosus* GG and *Bifidobacterium animalis* subsp. *Lactis* BB-12 were purchased from Chr. Hansen Holding A/S (Horsholm, Denmark). Biocodex (France) supplied *Saccharomyces boulardii* CNCM I-745. A control test was used to confirm the viable number of probiotic strains. For the first two probiotics: *Lactobacillus rhamnosus* GG and *Bifidobacterium animalis* subsp. *Lactis* BB-12, water samples were controlled by inoculation on Man, Rogosa and Sharpe agar medium in anaerobic conditions. They were found to contain a final dose of 1 × 10^10^ and 1 × 10^9^ cfu/mL, respectively. Those of *Saccharomyces boulardii* CNCM I-745 were counted on Rose Bengal Chloramphenicol agar, and the final dose was 3 × 10^9^ cfu/mL.

The animal groups of this trial were organised as follows:

(i) Group 1: (C) (*n* = 10): control group, rabbits with none of the probiotics.
(ii) Group 2: (BA) (*n* = 10); rabbits given 1 × 10^9^ cfu/mL of *Bifidobacterium animalis* subsp. *Lactis* BB-12 throughout the experiment following a previous protocol [18].
(iii) Group 3: (LR) (*n* = 10); rabbits given 1 × 10^10^ cfu/mL *Lactobacilllus rhamnosus* GG throughout the experiment following a previous protocol [19].
(iv) Group 4: (SB) (*n* = 10); rabbits given 3 × 10^9^ cfu/mL of the yeast *Saccharomyces boulardii* CNCM I-745 throughout the experiment following a previous protocol [20].

From day 31, the probiotic intake was stopped and all the rabbits were fed until day 60 with only the basic feed [19]. The purpose of this interruption on day 30 is to test the persistence of the influence of these probiotics on the same parameters after the suspension of the supplementation.

### 2.2. Evaluation of the Blood Parameters

The blood samples were drawn from all the rabbits by puncture of the auricular vein following previous protocols [21] after 12 h of fasting [22] on day 0, day 15, day 30, day 45 and day 60. The blood, carefully sampled, was distributed into two different types of tubes that contain anticoagulants (EDTA, heparin).

#### 2.2.1. Haematological Parameters

For the determination of certain haematological parameters such as red blood cells (RBC), haemoglobin (HGB), hematocrit (Ht), white blood cells (WBC), lymphocytes (LYMPHO≠); monocytes (MONO≠), neutrophils (NEUT≠), eosinophils (EO≠) and basophils (BASO≠), EDTA (EthylDiamineTetrAcetate) tubes were used. After sampling, the tubes were placed in a cooling box that contains wet ice and subsequently transported to the laboratory for further analyses. The values of the various haematological parameters were estimated by an automatic analyser (Shenzhen Mindray BC3000 plus, China) following previous protocols [23].

#### 2.2.2. Biochemical Parameters

The biochemical assays were performed using Heparin tubes for the measurement of the biochemical and ionic parameters. These included glucose (GLU), total cholesterol (TC), high-density lipoprotein (HDL), triglycerides (TG), total proteins (TP), albumin (ALB), urea, creatinine (CREA), alanine aminotransferase (ALT), aspartate aminotransferase (AST), iron (Fe), calcium (Ca), phosphorus (P), sodium (Na) and potassium (K).

The collected blood samples were stored first in a cooler box, then centrifuged (1500 rpm/15 min) to collect the plasma. Subsequently, the plasma was extracted and stored at −20 °C until analysis. The assays were performed using an automatic analyser AUTOLAB AMS (Analyser Medical System; Paris, France) as recently described [16]. Sodium and potassium were determined using a semi-automatic spectrophotometer: WP21B (Genius, Shenzhen Genius Electronics Co., Ltd., Shenzhen, China) as previously described [24].

### 2.3. Weight Measurement over Time and Growth Performance Parameters

The live body weight of the rabbits was determined by measuring their weight weekly in the morning at the same time by means of a digital scale until the end of the trial as described in earlier studies [16,25]. The feed intake of each group of rabbits per day was determined following previous protocols [26]. Then, the Feed Conversion Ratio (FCR) was calculated according to the following formula: FCR = daily feed intake (g)/daily weight gain (g).

### 2.4. Statistical Analyses

The data were analysed by means of XLSTAT 2018.1.1 (AddinSoft, Paris, France) using a general linear model that considered the main effects of this study (group, sex, time and their interactions). This model tested the fixed effects of the group (C (control), BA (rabbits with the probiotic *Bifidobacterium animalis* subsp. *Lactis* BB-12), LR (rabbits with the probiotic *Lactobacillus rhamnosus* GG) and SB (rabbits with the probiotic *Saccharomyces boulardii* CNCM I-745)), sex (female and male) and time (0, 15, 30, 45 and 60 days) following an ANOVA with repeated measures.

All the interactions were considered for the biochemical, haematological parameters and the body weight gain after the probiotic supplementation. Least squares means (LSmeans) were generated for all the interactions and factors considered in this trial. The results after post hoc Turkey test comparisons, further confirmed with the Bonferroni test, were considered significant at the level of 5% (*p <* 0.05). The blood parameter values and weight were presented as Least squares means ± standard error of the means (sem).

## 3. Results and Discussion

**Figure 1:**
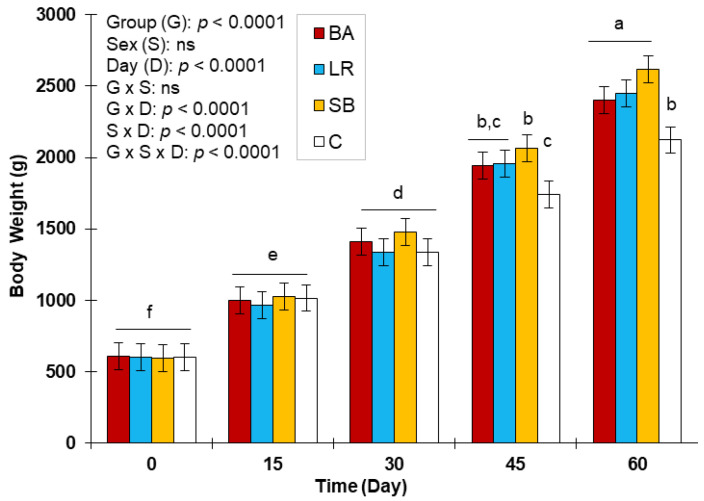
Effects of dietary supplementation with probiotics on live body weight (g) of healthy rabbits. (C): control group, (BA): *Bifidobacterium animalis* subsp. *Lactis* BB-12 group, (LR): *Lactobacillus rhamnosus* GG group, (SB): *Saccharomyces boulardii* CNCM I-745 group. LS-means (±SD) with different superscripts (a, b, c, d, e, f) are significantly different (*p* < 0.05).

The results of this trial showed an improvement in the biochemical and haematological parameters as well as in the weight of the rabbits from the 15th day after the administration of the three investigated probiotics with higher effect on the 30th day (Table 1, Table 2 and Table 3 and Figure 1). More specifically, a significant decrease was observed in the level of fasting glucose (*p* ˂ 0.001), total cholesterol (*p* ˂ 0.01), triglycerides (*p* ˂ 0.05), sodium content (*p* ˂ 0.01) and a significant increase in plasma levels for the total proteins (*p* ˂ 0.001), albumin (*p* ˂ 0.001), urea (*p* ˂ 0.05), iron (*p* ˂ 0.001), calcium (*p* ˂ 0.001) and potassium (*p* ˂ 0.05). In addition, a significant increase (*p* ˂ 0.001) in the number of red blood cells, haemoglobin level, total number of white blood cells and the absolute number of neutrophils was observed for the group of animals treated with *Lactobacillus rhamnosus* GG (LR), mainly at the 60th day (cessation of the treatment), which corresponds to the last day of the trial. Likewise, body weight increased significantly regardless of the used probiotic strain to supplement the diet. However, it seemed that the group treated with *Saccharomyces boulardii* CNCM I-745 (SB) had the highest values (2616.5 vs. 2122.2 g) and the lowest value of FCR compared to the controls (C) (2,36 vs 3,77). Regarding the group supplemented with *Bifidobacterium animalis* subsp. *lactis* BB-12, despite the improvement generated by this strain, the highest values were observed for the potassium level compared to the other groups. Interestingly, no significant difference was observed among the three probiotic strains. On the other hand, significant differences between males and females and among the different sampling times (day 0, 15, 30, 45 and 60) were detected for some parameters. The detailed results of the blood parameters and body weight among the four groups of rabbits during the 60 days of the experiment are reported in Table 1, Table 2 and Table 3 and Figure 1.

### 3.1. Biochemical Parameters

#### 3.1.1. Fasting Glucose Level

A significant decrease (*p <* 0.001) in the fasting glucose level was found for the three groups supplemented with the three probiotics compared to the control, and this started from the 15th day of the treatment (Table 1 and Figure S1). More specifically, the lowest values of the glucose level were detected in the group supplemented with *Saccharomyces boulardii* CNCM I-745 (SB) between the 30th and 45th day of treatment (0.93 ± 0.12 g/L and 0.94 ± 0.09 g/L), respectively (Figure S1). In addition, even after stopping the administration of the three probiotics on day 60, the fasting glucose values of the three treated groups were lower compared to the control (Figure S1). Interestingly, no significant difference (*p* ˃ 0.05) was observed between the two sexes (Table 1). The results obtained in this study confirmed the findings of previous works, demonstrating the beneficial effects of probiotics, in particular their ability to manage blood sugar in animal models [16,27,28,29,30]. Moreover, the positive effect of *Saccharomyces boulardii* CNCM I-745 was observed on type 2 diabetes in a previous study that used the same strain in mice for 4 weeks [28]. These results can be partly explained by the changes in the composition of the intestinal microbiota after the treatment with this yeast. Further, these changes could also be related to the host’s energy metabolism response. In another study using mice db/db, the treatment with *Lactobacillus rhamnosus* GG at 108 cfu for 4 weeks was found to adjust the blood glucose levels in the diabetic animals, mainly by reducing endoplasmic reticulum stress and suppressing the activation of macrophages, which results in an increased insulin sensitivity [31]. However, it is worthy of considering that a hypoglycemic effect of many *Bifidobacterium* spp. strains might induce adipocytes differentiation into a cell type capable of generating insulin sensitivity [32]. The supplementation of rabbits during 21 days with a combination of 109 cfu/mL of *Enterococcus faecium* CCM7420 and 30 g/kg feed from the bacteriocin extract of *Eleutherococcus senticosus* showed a significant reduction of fasting blood glucose compared to the controls [33]. Although, the beneficial effect of different probiotics on blood glucose regulation has been shown by several studies, some of them have found a hyperglycemic effect of certain probiotic strains compared to untreated animals. This can be exemplified by an earlier study that used rabbits treated with *Lactobacillus acidophilus* (*Mega acidophilus*) with a dose of 2 × 10^8^ cfu/kg weight/day during a period of 4 weeks [34]. Thus, the hypoglycemic effect of these probiotics could have a positive impact on the prevention of type 2 diabetes in animals and humans, in agreement with other studies [35].

#### 3.1.2. Lipids Parameters

The total cholesterol and triglycerides levels were found to be significantly lower (*p* ˂ 0.01) in the three groups supplemented with the three probiotics compared to the controls (Table 1). This decrease was recorded from the 15th day of treatment and particularly for the SB group (*p* ˂ 0.05). The lowest values were observed on the 30th day for total cholesterol (0.40 ± 0.12 g/L) (Figure S2) and between the 15th and 45th day for triglycerides (0.26 ± 0.04 g/L) (Figure S3). Regarding HDL, no significant difference was observed (Table 1). After stopping the treatment (from day 31 to day 60), a stabilisation of total cholesterol and triglyceride levels between the 30th and 45th day followed by a slight increase was found for the three treated groups (Figures S2 and S3). Furthermore, a significant difference between males and females was noted for triglycerides in the whole experiment (*p* ˂ 0.01), in particular for the males, who exhibited higher values than females (Table 1). Interactions (group × sex) for total cholesterol (*p* ˂ 0.01) and for triglycerides (*p* ˂ 0.001) were noticed (Table 1).

The positive effect of probiotics in improving the lipid profile in rabbits has been reported in certain studies. For example, a recent study reported a significant decrease in total cholesterol levels and triglycerides in rabbits fed with *Lactobacillus plantarum* (*Lactiplantibacillus plantarum*) at 10^6^ cfu/g for 8 weeks [36]. The lowering effect of cholesterol by *Sacharomyces boulardii* CNCM I-745 has been reported recently in hamsters treated with 3 g/kg for 21 days [20]. Another study by our group on obese rabbits supplemented with a mixture of *Lactobacillus plantarum* 299 v (10^10^ cfu/mL) and *Bifidobacterium animalis* subsp. *lactis* BB-12 (10^9^ cfu/mL) for 30 days, showed a significant decrease in cholesterol and triglyceride levels [16]. Hypercholesterolemic adult animals experienced a significant decrease in total cholesterol levels after consuming a yoghurt containing 5 × 10^9^ cfu/g of active *Lactobacillus reuteri* NCIMB 30242-BSH (probiotic capable of releasing Bile Salt Hydrolase-type enzymes) twice a day for 6 weeks [37]. These authors did not report any effect on triglycerides compared to another work that used human volunteers that consumed during 5 weeks a yoghurt rich in *Bifidobacterium animalis* subsp *lactis* DGCC 420 (*B. lactis* 420) (3.0 × 10^6^ cfu/g) and *Lactobacillus acidophilus* 74-2 (9.3 × 10^8^ cfu/g) [38]. It seems from the large literature that certain microorganisms present in probiotic additives could assimilate the cholesterol present in the gastrointestinal tract for their own cellular metabolism [39]. In fact, earlier studies have shown that a probiotic can lower serum cholesterol by inhibiting hydroxymethyl-glutaryl-coenzyme A, which is an enzyme involved in the cholesterol synthesis pathway [40]. The results of this study confirm those of previous studies revealing that the lipid profile is sex dependent [41], as in this study males presented significantly higher levels compared to females

Overall, these preliminary results in the improvement of the lipid profile, as well as of the glycaemia, could bring a particular advantage in the research carried out for the treatment and the prevention of the metabolic syndrome especially when using rabbit as an experimental animal model. This could be further considered as an alternative strategy for the prevention of cardiovascular diseases [42].

#### 3.1.3. Total Proteins and Albumin Contents

The albumin results were found to change in this study and significantly increased (*p* ˂ 0.001) in the groups treated with the three probiotics compared to the controls (Table 1). Similarly, a significant difference (*p* ˂ 0.001) was observed for all groups (C, SB, BA, LR) for the total proteins from day 0 to day 60 (Table 1). The group SB had the highest total proteins values from the 30th day (81.52 ± 20.95 g/L (SB) vs. 53.95 ± 18.87 g/L (C)) and a rate of 43.54 ± 10.65 g/L (SB) vs. 29.44 ± 11.42 g/L (C) for albumin (Figure S4). After stopping the probiotic treatment (from day 31 until day 60), a slight decrease in total protein and albumin levels for both SB and BA groups was observed on the 45th day, while the total protein values of the two groups LR and C were almost similar to that of the 45th day. Further, the rates stabilised between the 45th and the 60th day for all groups (Figures S4 and S5). A significant difference (*p* ˂ 0.001) between the two sexes was noted in both total protein and albumin contents, where higher values were observed for males (Table 1).

Overall, the improvement in total protein levels indicates that the probiotics might have a beneficial effect on protein metabolism, which is closely related to the improvement in body weight in the animal groups that were fed with the probiotics. These results agree with a previous study that demonstrated a considerable increase in the plasma levels of total proteins and albumin in a New Zealand rabbit breed following a supplementation with *Saccharomyces cerevisiae* for a period of 8 weeks [43]. In line with our findings, a study that used *Lactobacillus rhamnosus* GG (10^9^ cfu/mL) in the feeding of rabbits for 14 days had total protein levels slightly higher than those of the control group [19]. Furthermore, an increase in the concentration of proteins and albumin was reported in a study that supplemented for 8 weeks rabbits with 10^6^ cfu/g of *Lactobacillus planetarium* [36]. The increase in plasma protein level could be explained by two reasons. First, this can be a consequence of a better absorption and utilisation of nutrients by the intestine [44]. Second, it can be related to the increase in the level of globulins, which constitutes a fraction of serum proteins, indicating a possible improvement in the immunity of rabbits, in particular when *Saccharomyces boulardii* CNCM I-745 is used [45]. Regarding albumin, which is the main protein component of serum, this fraction can exert an effect on the humoral response and is likely to support the increase in immune organs after probiotic treatment [46,47]. The sex effect observed for these two parameters, mainly with higher amounts for males compared to females, is in contrast to those reported in the literature [48]. Another study demonstrated no sex effect in Angora breed rabbits [49]. Although further studies are needed to better understand this disparity among studies, we think that time can be a driver of these differences as a consequence of the growth of the animals as reported in other studies [50]. Thus, probiotics have participated in the assimilation of food proteins, which might partly explain the high level of plasma proteins.

#### 3.1.4. Urea, Creatinine, ALT and AST

No significant difference was observed between the four groups for creatinine, ALT and AST (*p* ˃ 0.05). Furthermore, a slight significant increase in urea was observed in the groups supplemented with the three probiotics (*p* ˂ 0.05) from the 30th day (Table 1 and Figure S6). However, from the 45th day of stopping the treatment, there was a slight decrease in urea values (Figure S6). A significant difference between the two sexes in favour of males was observed for urea (*p* ˂ 0.001) and creatinine (*p* ˂ 0.01). Thus, urea is the only parameter among the four that showed a significant difference over time (*p* ˂ 0.01) throughout the experimental period (Table 1). It has to be noted that group × sex interaction was observed for AST (*p* ˂ 0.001). The values were higher for females in the groups SB and LR, unlike the BA and control groups, where males had greater values (Table 1). Increased uremia accompanied by hypercreatininaemia is among the preclinical signs of renal damage [51]. The slight increase in uremia caused by probiotics could be due to the increase in total protein concentrations of serum, being aware that urea is a metabolite resulting from protein catabolism in the liver. Indeed, a positive correlation between these two parameters has been described [52]. The gender and administration time effects of probiotics, which systematically follow those of total proteins, support this hypothesis. Some studies have shown that probiotics could decrease urea levels, particularly in subjects with chronic kidney disease. For example, a study using human volunteers presenting chronic kidney disease, urea levels were found to be less than the control group after receiving 16 × 10^9^ cfu of *Lactobacillus caseishirota* during 8 weeks [53]. The concentration of creatinine, a degradation product of muscle creatine, depends essentially on the amount of muscle mass, and the kidney’s ability to perform its filtration [54]. These physiological data explain the influence of sex on the variation of this parameter. The males, who have greater muscle mass, also have higher values than the females. Probiotic supplementation has no significant effect on the plasma concentrations of creatinine, ALT and AST. Our results are in agreement with those of an earlier study that used healthy New Zealand rabbits treated with 0.4 g/kg of a mixture of two probiotics (*Bacillus subtilis* and *Bacillus licheniformis*) for 8 weeks [46]. The treatment with this mixture of probiotics may have a beneficial effect on lowering ALT and AST levels after liver damage caused by *Pasteurella multocida* infection. Overall, it seemed that the probiotics used in this trial have little negative effects on liver and kidney function.

#### 3.1.5. Iron, Calcium, Phosphorus, Sodium and Potassium

A significant increase (*p* ˂ 0.001) in plasma iron and calcium levels was observed in this trial from day 15 in the groups treated with the three probiotics compared to the control (Table 2). The highest levels were observed in the LR group (iron: 1.98 ± 0.28 mg/L and calcium: 94.18 ± 12.73 mg/L, at the 45th and 60th days of cessation treatment, respectively (Figures S7 and S8). For sodium, a significant decrease (*p* ˂ 0.01) was observed in the treated groups compared to the control (Table 2). More specifically, the SB group has the lowest values since the 15th day (Figure S9). In addition, a weakly significant increase (*p* ˂ 0.05) in serum potassium was observed, with the highest concentrations in the BA group mainly from the 30^th^ day to the end of the experiment (cessation of the treatment) (Figure S10), while no significant difference was observed for phosphorus (*p* ˃ 0.05). A sex effect on iron, calcium, sodium and potassium levels was perceived (*p* ˂ 0.01) (Table 2) with the highest calcium amounts noticed in females.

It resulted from this study that the probiotics could have beneficial effects on mineral status, especially on calcium and iron elements. The gut microbiota is known to have a primary role in regulating mineral absorption and is considered as a new pathophysiological regulator of intestinal iron absorption [55]. In this context, diabetic subjects treated for 8 weeks with a mixture of several probiotic strains exhibited higher calcium levels. A study by Hoppe and co-workers reported that the intake of *Lactobacillus plantarum* 299 v at 10^9^ or 10^10^ for 4 days by human volunteers increased iron absorption by about 50% [56]. Another study evidenced the influence of soy yoghurts enriched with *Bifidobacterium lactis* Bb-12 or *Bifidobacterium longum* Bb-46 for 45 days, on the bioavailability of Ca, P and Zn and bone mineralisation in rats [57]. In this study, the authors showed that the rat serum contents of Ca and P were approximately double in comparison to the control. The findings of this trial are also in line with those of Lollo and co-workers, who determined the beneficial effects of a cheese rich in probiotics on the parameters of hypertension in hypertensive rats [58]. The authors also observed a significant decrease in serum sodium levels against a significant increase in potassium. Taken all together, the improvement in the absorption of these ions at the cecum level could be the result of increased fermentation during probiotic processing, which promotes fermentation of carbohydrates and production of short chain fatty acids, particularly acetate, propionate and butyrate [59]. Regarding the influence of gender on calcium, our results disagree with a recent study that reported no gender effect [60]. Similarly, another study has shown that sex has no effect on calcium and potassium [48]. For serum iron, there were some very earlier studies that identified a difference between male and female rabbits [61]. The significant changes in iron, calcium and sodium levels throughout the experiment could be the result of increased mineral requirements during the growth period, which is accompanied by changes in ionic metabolism [62]. Finally, the group × sex interaction observed could be explained by a sexual dimorphism of the intestinal microbiota in the males and females, and therefore, the microorganisms of the probiotics are not acting through the same pathways [63,64]. Ultimately, the probiotics used in this study showed a beneficial effect on the level of iron, which enters into the composition of haemoglobin and therefore in the prevention of anaemia [65]. In addition, improving the calcium level could have an impact on the prevention of hypocalcaemia, especially in rabbits, which have a particular metabolism [66].

### 3.2. Haematological Parameters

#### 3.2.1. Red Blood Cells, Hemoglobin and Haematocrit

For the number of red blood cells (RBCs) and the level of haemoglobin (HGB) in the plasma of the rabbits that received the three probiotics, a significant increase (*p* ˂ 0.001) was found compared to the controls (Table 3) and the highest values were for the LR group on day 60 (cessation of the treatment) (Figures S11 and S12). For haematocrit (HCT), no significant difference was detected among the three groups (Table 3). However, a significant difference was observed between the males and females for both RBCs and HCT, while the females have the highest rates of RBCs unlike HCT (Table 3). Over time, differences in the values of RBCs and HGB during the different sampling days were observed (Table 3). Interactions between group × sex and group × day were found for HGB (Table 3), between group × day and sex × group × day (*p* ˂ 0.01) for RBCs, and no interaction for HCT (Table 3).

In this study, noticeable improvement in the erythrogram (a graphical representation of RBCs) was evident for all three investigated probiotics. This could be in part due to the effects of the probiotic strains on the balance of the nutrient profile and their antioxidant activities [43]. The influence of probiotics on certain haematological parameters of rabbits has been demonstrated by several authors [26,46,67,68]. Some studies related this to the stimulation of the haematopoietic organs [69] and also to the indirect effect of some lactic acid bacteria, including *Lactobacilli*, by increasing the bioavailability of dietary iron through several mechanisms such as the reduction of intestinal pH [70]. In agreement with our findings, the use of *Lactobacillus rhamnosus* for a period of three months in combination with other probiotic strains (*Lactobacillus acidophilus*, *Lactobacillus casei*, *Lactobacillus bulgaricus*, *Bifidobacterium breve*, *Bifidobacterium longum*, and *Streptococcus thermophilus*), increased the level of haemoglobin remarkably [71]. In contrast, other studies have noted that the treatment with *Pediococcus acidilactici* at a concentration of 10^9^ cfu/g does not affect the erythrogram of broilers [72]. Nevertheless, it has to be emphasised that other factors could increase the number of red blood cells such as the response to cold stress [73] or dehydrated animals [74].

Our results identified a sex effect on RBCs and HCT in accordance with earlier studies [75]. Meanwhile, it is important to note that the sex effect is not always observed among studies that used rabbits [76], which can be, for instance, related to breed, age, diet and environment. It was noted that there is a significant group × day interaction for RBC and HGB. However, there are no previous studies showing the interaction of the influence of probiotics over time, especially after the discontinuation of the administration of the probiotics. Plasma iron level recorded in the treated groups, in particular for the LR group, demonstrated an improvement in the erythrogram during this experiment, with the improvement over time in plasma iron levels, which is the basic constituent of haemoglobin. Regarding the group × sex interaction for HGB that highlighted higher values for the male rabbits of LR and SB groups, for the BA group, it concerned mainly the female rabbits. This difference can be related to the mechanism of action of each probiotic strain and to the targeted microbiota groups [67]. RBCs was the only blood parameter for which a sex × group × day interaction was observed, which could be a consequence of the process of synthesising red blood cells from the proliferation of haematopoietic stem cells up to the acquisition of its final properties [77].

#### 3.2.2. White Blood Cells, Lymphocytes, Monocytes, Neutrophils, Eosinophils and Basophils

A significant increase was observed for the total number of white blood cells (WBC) and the absolute number of neutrophils (NEUT≠) in the rabbits that were fed with the three probiotics (*p* ˂ 0.001) (Table 3). The LR group recorded the highest values compared to those of WBC (Figure S13) and NEUT≠ (Figure S14). No significant difference was found for lymphocytes (LYMPHO≠), monocytes (MONO≠), eosinophils (EO≠) and basophils (BASO≠) among the four groups (Table 3). The values of WBC and NEUT≠ were affected by sex (Table 3). In parallel, significant differences during the sampling period were observed for WBC, NEUT≠, EO≠ and BASO≠ (Table 3). However, we observed some significant interactions within group × sex for LYMPHO≠, NEUT≠ and BASO≠ and a group × day interaction for WBC and NEUT≠ (Table 3).

The modulation of the host’s immune system is one of the key properties of probiotics [78] and white blood cells have an important role in innate or nonspecific immunity (phagocytosis, pro-inflammatory cytokines), which can be modulated by probiotics. For example, it was previously demonstrated that rabbits receiving *Lactobacillus rhamnosus* GG had greater values of WBC compared to those receiving the same dose of *Lactobacillus plantarium* and *Lactobacillus reuteri* [67]. An increase in WBC could indicate a relatively lower susceptibility to different diseases [46]. Our results are also consistent with previous findings showing an increase in the total number of white blood cells and neutrophils using probiotics [26], with a key role in the orchestration of immune responses [79]. Another study on mice showed that *Lactobacillus rhamnosus* GG has a very good capacity for adhesion to intestinal epithelial cells accompanied by a modulation of the immune system [80], which may explain in our trial the high values of some parameters, mainly at the 60th day of stopping treatment. It was also previously demonstrated that *Bifidobacterium animalis* subsp. *lactis* BB-12 has immunomodulatory properties and anti-inflammatory effects in the case of people treated for a period of 4 weeks [81]. The interaction (group × sex) for lymphocytes, neutrophils and basophils could be related to the specificity of the microbiota of each sex [82]. Accordingly, a study on piglets treated with *Bifidobacterium lactis* NCC2818 evidenced an effect on the development of the immune system, but in a sex-dependent manner [64]. The group × day interaction on WBC and neutrophils could be explained by the development of the immune system with age [83] and the administration of probiotics at the same time in this trial. Most probiotics stimulate the innate immune defences (phagocytosis, pro-inflammatory cytokines) and act positively on the duration of infectious episodes, in particular neutrophils, which play a key role in the immune response. In addition, a beneficial anti-inflammatory effect has also been observed in certain pathological situations [78]. The mechanisms involved in these beneficial effects are currently the subject of numerous studies [84]. In addition, the neutrophil/lymphocyte ratio is a marker that reflects the state of systemic inflammation or oxidative stress [85].

It is noted that even after interruption of the treatment between the 30th and 60th days of the experiment, the three probiotic strains continue to show beneficial effects on the certain parameters investigated in this trial, in particular for the two lines; red and white as well as plasma levels of iron and calcium. These long-term effects could constitute a biological tool against certain deficits, which makes it possible to improve the blood count. Indeed, the effect of probiotics even after discontinuation of treatment could have a relationship with the persistence and long-term viability of these strains in the gastrointestinal tract as evidenced in earlier studies [19].

### 3.3. Evolution of Body Weight and Feed Conversation Ratio with the Three Probiotics Supplementation

In Figure 1, the evolution of the weights of the rabbits is shown for each probiotic group compared to the controls. Significant differences (*p* ˂ 0.0001) among the groups treated with the three probiotics compared to the controls were observed, and regardless of the treatment, all groups had higher values. It seemed that the differences were more important from the 30th day of the treatment. On the 60th day (cessation of the treatment), the SB group had the highest weight (2616.5 ± 184 g) followed by LR (2448.5 ± 115 g) and BA (2401.8 ± 71 g) compared to the controls that had the lowest final weight (2122.2 ± 66 g). In this trial, sex had no effect on the body weight of the rabbits (*p* ˃ 0.05). As expected, a time effect showed a significant impact on the weight over the experimental time (Figure 1). In addition, the results of Table S4, confirm that the three groups supplemented with the three strains of probiotics presented lower consumption indices compared to the control group (C: 3.77 versus BA: 2.91; LR: 2.92 and SB: 2.36) (*p* ˂ 0.001), while the SB group had the lowest value. In addition, this last batch showed the highest daily gain (*p* ˂ 0.001) compared to the other groups: 33.72 g (SB) vs. 30.8 g (LR) vs. 29.78 g (BA) vs. 25.25 g (C). It is well accepted that FCR is a good indicator used to express the efficiency of converting feed into body weight gain [86]. Indeed, any decrease in FCR as well as a decrease in feed consumption in link with the variation in the average daily gain could reflect the success of breeding from a zootechnical and economic point of view [87]. The study carried by Ezema and Eze [26] found that probiotics at 0.12 g/kg diet in rabbit feed enhance the growth performance, digestibility of nutrients, and feed efficiency of the animals. It was also found that probiotics are able to modify the gut microflora [88], hence improving the productive performances of rabbits. The underlying mechanisms might be various; for instance, they can be related to the stimulation of intestinal enzyme production, stimulation of the immune system of the host, the reduction of toxin production, increased resistance to colonisation, and reduction of stress in rabbits [89,90].

Many studies aimed to identify alternatives to the use of antibiotics in the objective of improving both the health and animals’ production performances [91]. The promoting of the risk of the resistance of bacteria towards antibiotics, and because of consumer demands for animal products without antibiotics, resulted in the agricultural industry using animal growth promoters in animals production intended for feeding. In a recent study by Bassiony et al. [92], an 8-week feeding experiment examined the influences of single or/and double strains of probiotics (*Enterococcus faecium* NCIMB 11181 and *Clostridium butyricum*) compared to antibiotic colistin on growth, haematological variables, blood serum metabolites and caecal fermentation in post-weaning New Zealand White rabbits exposed to heat stress conditions. The authors demonstrated that the supplemental *Enterococcus faecium* NCIMB 11181 and *Enterococcus faecium* NCIMB 11181 and *Clostridium butyricum* together enhanced rabbit growth and feed utilisation, while improving biochemical and immunological indicators. Therefore, it seemed that probiotic supplements could be used as alternatives to antibiotics to promote the growth of rabbits even under heat stress conditions. Further studies are needed to test our conditions and the three probiotics we targeted in the production of the ITELV2006 rabbit strain with or without the use of antibiotics.

The findings of this trial also agree with the current knowledge and impact of probiotics as growth promoters on rabbits. For example, feed supplementation with *Saccharomyces cerevisiae boulardii* CNCM I-1079 between 37 to 84 days of age in New Zealand rabbits has beneficial effects on growth parameters [93]. Another earlier study reported that the body weight of rabbits increased by 3 to 6% after supplementation with a mixture of *Streptococcus faecium*, *Lactobacillus acidophilus*, protease, amylase, cellulase and *Saccharomyces* yeast, compared to untreated animals [94]. Furthermore, the addition of 0.05% of a mixture of probiotics (*Lactobacillus bulgaricus*, *L. acidophillus*, *L. helveticus*, *L. lactis*, *Streptococcus thermophillus* and *E. faecium*) increased the average daily gain by almost 12% in New Zealand rabbits [95]. Similarly, a supplementation with *Lactobacillus acidophilus* (0.8 × 10^9^ cfu/g) was found to increase the final weight of the rabbits compared to the controls [96]. The beneficial effect of probiotic supplementation on growth parameters can be related to several factors. For example, it can be due to the improvement of digestion and intestinal absorption of nutrients following a restoration of the balance of the intestinal microflora, which in turn plays a pivotal role in the integrity of the intestinal mucous barrier and the digestive and immune functions [97]. Our results agree with those of Yalçin and co-authors who observed that the final weight was similar between males and females [98], while other studies reported opposite results namely for females that had higher body weight when treated with probiotics [99]. The influence of the experimental period on body weight can be explained by the rapid growth of rabbits during the period of the trial, which was carried out between the age of weaning and slaughter. Nevertheless, the group × day interaction could be due to the changes in the intestinal ecosystem of the rabbits that might be induced by the probiotics or other factors influencing the composition of gut microbiota such as age or quality of the feed [6]. Overall, unlike the beneficial effect of the three investigated probiotic strains on weight gain under physiological conditions, other strains could have a controlling role in animals or in obese people who developed a metabolic syndrome [16,100,101].

## 4. Conclusions

Supplementing the feed of ITELV2006 strain rabbits with *Lactobacillus rhamnosus* GG, *Bifidobacterium animalis* subsp. *Lactis* BB-12 and *Saccharomyces boulardii* CNCM I-745 revealed beneficial effects on blood parameters of both red and white cells as well as on the final body weight and on the FCR of the rabbits. The group of rabbits treated with *Saccharomyces boulardii* CNCM I-745 presented the most relevant results concerning the biochemical constants such as the decrease in fasting glucose even after stopping the administration of probiotics between the 30th and the 45th day. Further, the lipid profile of the rabbits was improved, mainly by a significant decrease in cholesterol content and triglycerides. The group treated with *Lactobacillus rhamnosus* GG presented values of erythrocytes, haemoglobin, leukocytes, neutrophils, calcium and iron higher compared to those of the other groups. Further, *Bifidobacterium animalis* subsp. *Lactis* BB-12 was effective in increasing potassium. Overall, the observed improvement owing to the use of the three probiotic strains could be an essential tool and a key strategy to modulate the composition of the intestinal microbiota and improve parameters of paramount importance, including the productivity and health of rabbits. These preliminary results highlighting improvements in fasting blood sugar, total cholesterol and triglyceride levels could bring a particular advantage in the research carried out for the treatment and the prevention of the metabolic syndrome and should be confirmed by further in-depth analysis, such as through metagenomics analyses to better understand how to modulate the gut microbiota for optimised outcomes. Moreover, it is meaningful to investigate in a targeted study the effect of the three strains on muscle growth and development and to explore the possible consequences on meat quality traits of the rabbits supplemented with the probiotics.

## Figures and Tables

**Table 1:** Effects of dietary supplementation with probiotics on biochemical parameters of healthy rabbits ^1^.

Groups ^3^	Parameters ^2^		GLU (g/L)		TC (g/L)		HDL (g/L)		TG (g/L)		TP (g/L)		ALB (g/L)		UREA (g/L)		CREA (mg/L)		ALT (UI/L)		AST (UI/L)	
		Sex	M	F	M	F	M	F	M	F	M	F	M	F	M	F	M	F	M	F	M	F
	Days																					
C	0		0.96	1.01	0.39	0.59	0.09	0.18	0.25	0.33	61.21 ^bc^	48.60 ^d^	34.06 ^b^	29.31 ^bc^	1.99 ^c^	2.27 ^b^	11.91	11.17	45.76	47.85	43.43	33.55
	15		1.10	1.10	0.47	0.60	0.01	0.16	0.31	0.29	62.15 ^bc^	47.74 ^d^	34.48 ^b^	27.04 ^bc^	2.31 ^b^	2.05 ^b^	12.52	11.59	42.52	48.55	46.98	31.54
	30		1.04	1.09	0.55	0.64	0.11	0.15	0.33	0.31	64.22 ^bc^	43.68 ^d^	34.40 ^b^	24.50 ^c^	2.34 ^b^	1.98 ^c^	12.57	11.36	44.52	48.95	46.47	30.97
	45		1.07	1.19	0.57	0.56	0.14	0.12	0.34	0.32	64.99 ^bc^	53.65 ^c^	33.64 ^bc^	28.00 ^bc^	2.31 ^b^	2.08 ^b^	13.02	11.62	45.00	46.63	46.06	30.07
	60		1.13	1.08	0.56	0.58	0.12	0.12	0.33	0.32	64.57 ^bc^	54.65 ^c^	34.33 ^b^	27.77 ^bc^	2.22 ^b^	2.11 ^b^	13.26	11.88	44.52	47.96	45.40	31.31
BA	0		0.99	1.04	0.52	0.47	0.14	0.12	0.35	0.24	63.22 ^bc^	46.74 ^d^	35.44 ^b^	26.38 ^c^	2.02 ^b^	1.91 ^c^	12.03	10.91	46.59	42.06	38.93	36.24
	15		0.95	1.03	0.56	0.44	0.14	0.10	0.35	0.26	67.40 ^b^	49.38 ^d^	36.75 ^b^	32.02 ^bc^	2.17 ^b^	1.98 ^c^	12.05	10.98	47.30	48.21	40.90	34.93
	30		0.96	0.98	0.56	0.46	0.20	0.18	0.35	0.26	90.01 ^a^	58.68 ^c^	48.82 ^a^	31.31 ^bc^	2.70 ^a^	2.36 ^b^	12.75	10.89	49.00	46.90	40.34	38.44
	45		0.98	0.96	0.56	0.49	0.18	0.17	0.35	0.24	88.31 ^a^	57.57 ^c^	49.99 ^a^	29.46 ^bc^	2.60 ^ab^	2.41 ^ab^	13.05	11.33	49.00	46.90	39.96	38.31
	60		0.99	0.96	0.59	0.52	0.16	0.15	0.36	0.25	88.51 ^a^	55.97 ^c^	48.16 ^a^	28.81 ^bc^	2.40 ^ab^	2.31 ^b^	13.39	11.75	49.34	46.18	40.80	38.36
LR	0		0.94	0.95	0.41	0.62	0.15	0.13	0.28	0.29	56.74 ^c^	54.07 ^c^	34.99 ^b^	29.91 ^bc^	2.43 ^ab^	1.85 ^c^	12.33	11.80	39.36	50.51	34.51	39.08
	15		0.96	1.07	0.51	0.49	0.16	0.17	0.29	0.31	66.47 ^b^	46.20 ^d^	35.85 ^b^	28.91 ^bc^	2.41 ^ab^	2.03 ^b^	12.32	11.77	42.57	48.70	36.33	39.75
	30		1.05	0.86	0.45	0.45	0.17	0.14	0.30	0.31	66.01 ^b^	50.42 ^c^	36.06 ^b^	27.59 ^bc^	2.25 ^b^	2.11 ^b^	12.36	11.66	43.93	48.45	35.96	39.68
	45		0.93	0.94	0.45	0.43	0.16	0.14	0.30	0.30	65.77 ^b^	51.24 ^c^	35.79 ^b^	29.16 ^bc^	2.25 ^b^	2.14 ^b^	12.44	12.37	43.93	48.45	36.40	40.96
	60		0.94	1.00	0.48	0.42	0.14	0.13	0.30	0.31	68.41 ^b^	51.19 ^c^	34.81 ^b^	27.90 ^bc^	2.21 ^b^	2.12 ^b^	12.89	12.48	44.71	47.32	35.73	44.03
SB	0		1.05	0.95	0.37	0.57	0.15	0.13	0.30	0.27	53.40 ^c^	56.84 ^c^	31.70 ^bc^	29.11 ^bc^	2.13 ^b^	2.40 ^ab^	12.11	12.99	45.92	47.15	31.38	46.11
	15		0.95	1.00	0.37	0.50	0.13	0.17	0.26	0.26	55.42 ^c^	59.36 ^c^	33.80 ^bc^	33.10 ^bc^	2.29 ^b^	1.91 ^c^	12.30	12.16	43.73	47.70	35.85	44.94
	30		0.92	0.97	0.36	0.45	0.12	0.15	0.28	0.25	94.59 ^a^	68.47 ^b^	49.14 ^a^	37.95 ^b^	2.90 ^a^	2.32 ^b^	12.35	12.31	44.34	43.36	36.58	44.78
	45		0.91	0.95	0.38	0.44	0.13	0.14	0.27	0.25	92.88 ^a^	65.77 ^b^	48.41 ^a^	45.80 ^a^	2.83 ^a^	2.29 ^b^	12.77	12.27	44.34	43.35	37.54	45.74
	60		1.01	0.94	0.37	0.50	0.13	0.15	0.27	0.32	89.50 ^a^	67.21 ^b^	46.18 ^a^	35.46 ^b^	2.61 ^ab^	2.35 ^b^	13.57	12.89	44.84	42.67	38.01	45.82
Effects ^4,5^	Group (G)		***		**		ns		*		***		***		*		Ns		ns		Ns	
	Sex (S)		ns		ns		ns		**		***		***		***		**		ns		Ns	
	Day (D)		ns		ns		ns		ns		***		ns		**		Ns		ns		Ns	
	G × S		ns		**		ns		***		ns		ns		ns		Ns		ns		***	
	G × D		ns		ns		ns		ns		ns		ns		ns		Ns		ns		Ns	
	S × D		ns		ns		ns		ns		ns		ns		ns		Ns		ns		Ns	
	G × S × D		ns		ns		ns		ns		ns		ns		ns		Ns		ns		Ns	

^1^ The details of the statistical analyses in terms of the group, sex and day effects on each parameter are given in the Supplementary Table S1. ^2^ (GLU): glucose, (TC): total cholesterol, (HDL): high-density lipoprotein, (TP): total protein, (ALB): albumin, (CREA): creatinine, (ALT): alanine aminotransferase, (AST): aspartate aminotransferase. ^3^ (C): control group, (BA): *Bifidobacterium animalis* subsp. *Lactis* BB-12 group, (LR): *Lactobacillus rhamnosus* GG group, (SB): *Saccharomyces boulardii* CNCM I-745 group. ^4^ Significance: * *p* < 0.05; **: *p* < 0.01; ***: *p* < 0.001; ns: not significant. ^5^ The variables in each column not followed with the same letter are different (*p* < 0.05).

**Table 2:** Effects of dietary supplementation with probiotics on ionic parameters of healthy rabbits ^1^.

Groups ^3^	Parameters ^2^		Fe (mg/L)		Ca (mg/L)		P (mg/L)		Na (mmol/L)		K (mmol/L)	
		Sex	M	F	M	F	M	F	M	F	M	F
	Day											
C	0		1.40 ^bc^	1.35 ^c^	60.50 ^c^	72.43 ^bc^	43.20	44.32	142.90	135.22	3.79 ^ab^	3.74 ^ab^
	15		1.48 ^abc^	1.36 ^c^	65.77 ^bc^	76.68 ^bc^	44.61	45.20	142.39	142.66	3.86 ^ab^	3.85
	30		1.48 ^abc^	1.48 ^abc^	66.07 ^bc^	79.46 ^bc^	45.67	48.50	144.78	140.71	3.93 ^b^	3.46 ^ab^
	45		1.51 ^abc^	1.71 ^abc^	68.54 ^bc^	80.42 ^bc^	48.52	44.99	144.62	140.48	3.87 ^ab^	3.91 ^ab^
	60		1.45 ^abc^	1.67 ^abc^	69.47 ^bc^	81.06 ^bc^	49.20	45.93	144.36	141.35	3.88 ^ab^	3.80 ^ab^
BA	0		1.61 ^abc^	1.20 ^c^	73.36 ^bc^	56.80 ^c^	43.54	43.81	144.19	137.09	3.81 ^ab^	3.69 ^ab^
	15		1.60 ^abc^	1.45 ^c^	83.51 ^b^	59.09 ^c^	47.12	45.72	142.21	140.79	4.03 ^a^	3.94 ^ab^
	30		1.76 ^abc^	1.59 ^abc^	91.22 ^a^	66.01 ^bc^	48.14	49.65	144.30	141.97	4.11 ^a^	3.99 ^ab^
	45		1.81 ^abc^	1.56 ^abc^	90.93 ^a^	67.88 ^bc^	48.30	51.31	144.05	141.31	4.04 ^a^	3.89 ^ab^
	60		1.79 ^abc^	1.56 ^abc^	91.88 ^a^	70.22 ^bc^	48.04	59.94	145.23	140.53	4.09 ^a^	3.92 ^ab^
LR	0		1.58 ^abc^	1.22 ^c^	70.36 ^bc^	63.05 ^c^	43.00	44.22	144.58	135.86	3.77 ^ab^	3.73 ^ab^
	15		1.92 ^abc^	1.48 ^abc^	78.88 ^bc^	71.53 ^bc^	42.98	47.50	143.77	133.27	3.89 ^ab^	3.91 ^ab^
	30		2.17 ^a^	1.62 ^abc^	93.70 ^a^	83.02 ^b^	45.18	47.38	141.84	135.62	4.10 ^a^	3.79 ^ab^
	45		2.18 ^a^	1.77 ^abc^	96.88 ^a^	88.00 ^b^	45.36	48.56	140.44	137.56	4.07 ^a^	3.82 ^ab^
	60		2.13 ^a^	1.75 ^abc^	96.52 ^a^	91.85 ^a^	45.66	48.96	138.49	139.78	4.04 ^a^	3.81 ^ab^
SB	0		1.31 ^c^	1.44 ^c^	66.52 ^bc^	66.74 ^bc^	43.00	45.22	140.48	138.46	3.82 ^ab^	3.72 ^ab^
	15		1.46 ^abc^	1.58	72.50 ^bc^	72.30 ^bc^	46.06	44.74	135.69	134.61	3.87 ^ab^	3.92 ^ab^
	30		1.73 ^abc^	1.75 ^abc^	81.60 ^bc^	84.83 ^b^	45.16	44.88	136.85	134.82	3.87 ^ab^	3.96 ^ab^
	45		1.75 ^abc^	1.74 ^abc^	82.45 ^bc^	86.36 ^b^	46.06	47.50	137.14	133.71	3.92 ^ab^	3.88 ^ab^
	60		1.71 ^abc^	1.80 ^abc^	82.22 ^bc^	87.34 ^b^	45.30	47.62	138.87	135.90	3.81 ^ab^	3.84 ^ab^
Effects ^4,5^	Group (G)		***		***		ns		**		*	
	Sex(S)		**		Ns		ns		**		**	
	Day (D)		***		***		ns		ns		**	
	G × S		***		***		ns		ns		ns	
	G × D		Ns		Ns		ns		ns		ns	
	S × D		Ns		Ns		ns		ns		ns	
	G × S × D		Ns		Ns		ns		ns		ns	

^1^ The details of the statistical analyses in terms of the group, sex and day effects on each parameter are given in the Supplementary Table S2. ^2^ Abbreviations: (Fe): iron, (Ca): calcium, (P): phosphorus, (Na): sodium, (K): potassium. ^3^ (C): control group, (BA): *Bifidobacterium animalis* subsp. *Lactis* BB-12group, (LR): *Lactobacillus rhamnosus* GG group, (SB): *Saccharomyces boulardii* CNCM I-745 group. ^4^ Significance: * *p* < 0.05; **: p < 0.01; ***: *p* < 0.001; ns: not significant. ^5^ The variables in each column not followed with the same letter are different (*p* < 0.05).

**Table 3:** Effects of dietary supplementation with probiotics on haematological parameters of healthy rabbits ^1^.

Groups ^3^	Parameters ^2^		RBC (10^6^/µL)		HGB (g/dL)		HCT (%)		WBC (10^3^/µL)		LYMPHO≠ (10^3^/µL)		MONO≠ (10^3^/µL)		NEUT≠ (10^3^/µL)		EO≠ (10^3^/µL)		BASO≠ (10^3^/µL) ^6^	
		Sex	M	F	M	F	M	F	M	F	M	F	M	F	M	F	M	F	M	F
	Days																			
C	0		4.51 ^c^	4.91 ^bc^	10.56 ^bc^	10.67 ^bc^	33.06	31.94	6.12 ^d^	7.10 ^bc^	5.16	4.98	0.30	0.27	1.43 ^bc^	1.31 ^c^	0.09	0.08	0.01	0.00
	15		4.88 ^bc^	4.61 ^bc^	10.59 ^bc^	10.64 ^bc^	32.98	32.62	5.90 ^d^	7.79 ^ab^	5.05	5.03	0.37	0.35	1.50 ^bc^	1.26 ^c^	0.04	0.06	0.00	0.00
	30		4.79 ^bc^	4.47 ^c^	10.57 ^bc^	10.62 ^bc^	33.16	32.02	6.07 ^d^	7.26 ^b^	5.06	4.80	0.43	0.29	1.54 ^bc^	1.39 ^c^	0.04	0.05	0.00	0.00
	45		4.75 ^bc^	4.88 ^bc^	10.55 ^bc^	10.85 ^bc^	33.10	32.16	6.11 ^d^	7.47 ^ab^	4.84	4.63	0.32	0.35	1.52 ^bc^	1.91 ^bc^	0.05	0.05	0.00	0.00
	60		4.59 ^bc^	4.86 ^bc^	10.67 ^bc^	10.84 ^bc^	33.42	32.06	6.07 ^d^	7.41 ^ab^	4.73	4.77	0.44	0.34	1.64 ^bc^	1.91 ^bc^	0.02	0.04	0.00	0.00
BA	0		4.26 ^c^	5.11 ^b^	10.69 ^bc^	10.53 ^bc^	33.68	31.26	6.27 ^d^	7.27 ^b^	5.24	4.77	0.28	0.24	1.39 ^c^	1.35 ^c^	0.04	0.12	0.00	0.01
	15		4.66 ^bc^	4.51 ^c^	10.80 ^bc^	10.50 ^c^	34.04	31.40	6.50 ^d^	7.65 ^ab^	5.49	4.62	0.32	0.47	1.58 ^bc^	1.52 ^bc^	0.01	0.10	0.00	0.00
	30		4.87 ^bc^	4.69 ^bc^	11.05 ^ab^	10.54 ^bc^	34.44	32.28	7.31 ^b^	7.39 ^bc^	5.02	4.78	0.39	0.40	2.94 ^b^	1.91 ^bc^	0.01	0.08	0.00	0.00
	45		4.85 ^bc^	5.37 ^bc^	10.72 ^bc^	10.90 ^ab^	34.64	32.18	7.25 ^b^	7.23 ^b^	5.05	4.78	0.43	0.39	2.26 ^b^	1.91 ^bc^	0.01	0.06	0.00	0.00
	60		4.85 ^bc^	5.37 ^bc^	10.93 ^ab^	11.06 ^ab^	34.66	32.22	6.90 ^c^	7.38 ^ab^	5.65	3.84	0.38	0.39	2.33 ^b^	1.81 ^bc^	0.01	0.06	0.00	0.00
LR	0		5.04 ^bc^	4.41 ^c^	10.12 ^c^	10.92 ^bc^	32.72	32.18	7.07 ^bc^	7.47 ^ab^	4.28	5.88	0.29	0.28	1.57 ^bc^	1.32 ^c^	0.06	0.11	0.00	0.00
	15		4.36 ^c^	4.96 ^bc^	10.14 ^c^	10.93 ^ab^	32.76	32.80	7.90 ^ab^	8.17 ^ab^	5.05	6.69	0.43	0.47	1.95 ^bc^	1.43 ^bc^	0.03	0.03	0.00	0.00
	30		4.73 ^bc^	6.54 ^a^	11.95 ^a^	11.96 ^a^	33.58	33.32	8.42 ^ab^	10.07 ^a^	6.04	6.07	0.50	0.46	3.22 ^a^	2.54 ^b^	0.03	0.3	0.00	0.00
	45		6.01 ^b^	6.35 ^b^	12.12 ^a^	12.29 ^a^	34.02	34.10	8.63 ^ab^	9.73 ^a^	5.46	5.76	0.45	0.87	3.48 ^a^	3.14 ^a^	0.00	0.02	0.00	0.00
	60		7.01 ^a^	7.00 ^a^	12.16 ^a^	12.24 ^a^	34.54	34.42	8.61 ^ab^	10.22 ^a^	5.07	5.83	0.42	0.40	3.38 ^a^	3.41 ^a^	0.01	0.02	0.00	0.00
SB	0		4.71 ^bc^	4.71 ^bc^	10.97 ^ab^	10.63 ^bc^	32.96	32.58	4.49 ^d^	7.09 ^bc^	5.02	4.97	0.29	0.25	1.37 ^c^	1.33 ^c^	0.09	0.09	0.00	0.00
	15		4.85 ^bc^	4.76 ^bc^	10.90 ^ab^	10.76 ^bc^	33.70	32.72	6.51 ^d^	7.38 ^ab^	4.28	6.03	0.30	0.38	1.56 ^bc^	1.39 ^c^	0.03	0.03	0.00	0.00
	30		5.02 ^b^	5.22 ^b^	10.95 ^ab^	10.91 ^ab^	34.54	32.94	7.53 ^ab^	8.13 ^ab^	4.64	6.21	0.38	0.38	2.01 ^b^	2.08 ^b^	0.03	0.02	0.00	0.00
	45		4.47 ^c^	5.62 ^b^	11.87 ^a^	11.22 ^ab^	33.58	33.58	7.62 ^ab^	8.10 ^ab^	4.62	6.13	0.38	0.36	2.13 ^b^	2.61 ^b^	0.02	0.03	0.00	0.00
	60		5.47 ^b^	5.46 ^b^	11.83 ^a^	11.47 ^ab^	34.36	33.82	7.36 ^ab^	7.88 ^ab^	4.42	6.34	0.36	0.36	2.01 ^b^	2.14 ^b^	0.03	0.03	0.00	0.00
Effects ^4,5^	Group (G)		***		***		ns		***		Ns		ns		***		ns		ns	
	Sex(S)		*		Ns		**		***		Ns		ns		**		ns		ns	
	Day (D)		***		**		ns		***		Ns		ns		***		**		ns	
	G × S		ns		**		ns		ns		**		ns		**		ns		ns	
	G × D		**		***		ns		*		Ns		ns		***		ns		ns	
	S × D		ns		Ns		ns		ns		Ns		ns		ns		ns		ns	
	G × S × D		**		Ns		ns		ns		Ns		ns		ns		ns		ns	

^1^ The details of the statistical analyses in terms of the group, sex and day effects on each parameter are given in the Supplementary Table S3. ^2^ Abbreviations: (RBC): number of red blood cells, (WBC): number of white blood cells, (HGB): haemoglobin, (HCT): haematocrit. (NEUT#): number of neutrophils, (LYMPH#): number of lymphocytes, (MONO#): number of monocytes, (EO#): number of eosinophils, (BASO#): number of basophils. ^3^ (C): control group, (BA): *Bifidobacterium animalis* subsp. *Lactis* BB-12group, (LR): *Lactobacillus rhamnosus* GG group, (SB): *Saccharomyces boulardii* CNCM I-745 group. ^4^ Significance: * *p* < 0.05; **: p < 0.01; ***: *p* < 0.001; ns: not significant. ^5^ The variables in each column not followed with the same letter are different (*p* < 0.05). ^6^ The values of BASO# (number of basophils) were below 1.4 × 10^−2^ in all the samples, ranging from 1.4 × 10^−2^ to 7.5 × 10^−17.^

## Data Availability

No data copyright issues.

## Supplementary Materials

The following are available online at https://www.mdpi.com/article/10.3390/biology10111194/s1, Figure S1: Effects of dietary supplementation with probiotics on blood fasting glucose level of healthy rabbits. Figure S2: Effects of dietary supplementation with probiotics on blood total cholesterol level of healthy rabbits. Figure S3: Effects of dietary supplementation with probiotics on blood triglycerides level of healthy rabbits. Figure S4: Effects of dietary supplementation with probiotics on blood total proteins level of healthy rabbits. Figure S5: Effects of dietary supplementation with probiotics on blood albumin level of healthy rabbits. Figure S6: Effects of dietary supplementation with probiotics on blood urea level of healthy rabbits. Figure S7: Effects of dietary supplementation with probiotics on blood iron level of healthy rabbits. Figure S8: Effects of dietary supplementation with probiotics on blood calcium level of healthy rabbits. Figure S9: Effects of dietary supplementation with probiotics on blood sodium level of healthy rabbits. Figure S10: Effects of dietary supplementation with probiotics on blood potassium level of healthy rabbits. Figure S11: Effects of dietary supplementation with probiotics on number of red blood cells of healthy rabbits. Figure S12: Effects of dietary supplementation with probiotics on number of blood haemoglobin level of healthy rabbits. Figure S13: Effects of dietary supplementation with probiotics on number of white blood cells of healthy rabbits. Figure S14: Effects of dietary supplementation with probiotics on number of neutrophiles of healthy rabbits. Table S1. Effects of dietary supplementation with probiotics on biochemical parameters of healthy rabbits (from Figure S1A–J). Table S2. Effects of dietary supplementation with probiotics on ionic parameters of healthy rabbits (from Figure S2A–E). Table S3. Effects of dietary supplementation with probiotics on haematological parameters of healthy rabbits (from Figure S3A–I). Table S4: Growth performance of the rabbits supplemented with the three probiotic strains.
