# Supplementary material for: Influence of Three Probiotics Strains, Lactobacillus rhamnosus GG, Bifidobacterium animalis subsp. Lactis BB-12 and Saccharomyces boulardii CNCM I-745 on the Biochemical and Haematological Profiles and Body Weight of Healthy Rabbits
Source: Biology (Basel). 2021 Nov 17;10(11):1194. doi: 10.3390/biology10111194 (PMC8615081; doi:10.3390/biology10111194)
Supplement: Supplementary file 1 [file biology-10-01194-s001.zip › biology-1431328-supplementary_Figures.pdf]

biology

MDPI

Supplementary data

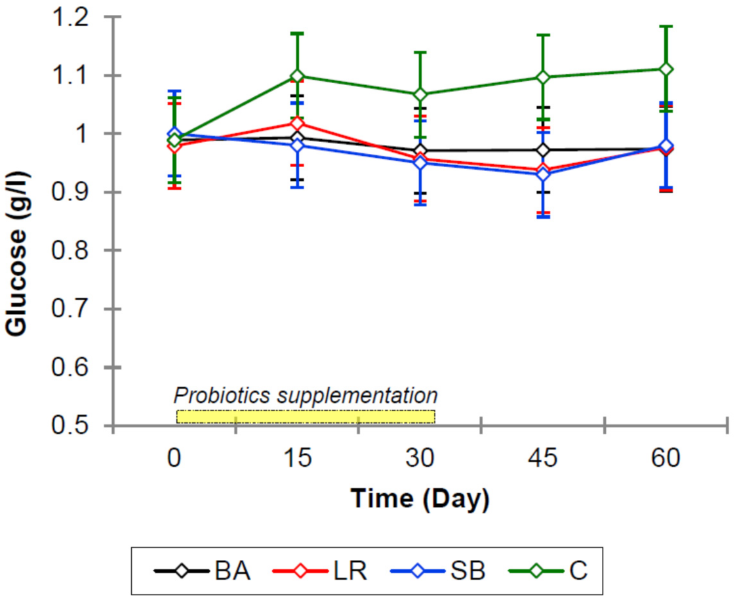

Figure S1. Effects of dietary supplementation with probiotics on blood fasting glucose level of healthy rabbits. (C): control group, (BA): *Bifidobacterium animalis* subsp. *Lactis* BB-12 group, (LR*): Lactobacillus rhamnosus* GG group, (SB): *Saccharomyces boulardii* CNCM I-745 group.

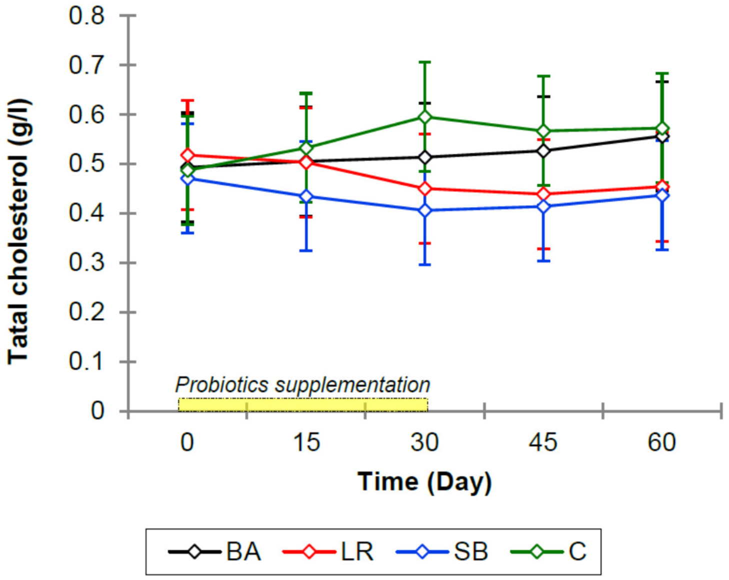

Figure S2. Effects of dietary supplementation with probiotics on blood total cholesterol level of healthy rabbits. (C): control group, (BA): *Bifidobacterium animalis* subsp. *Lactis* BB-12 group, (LR*): Lactobacillus rhamnosus* GG group, (SB): *Saccharomyces boulardii* CNCM I-745 group.

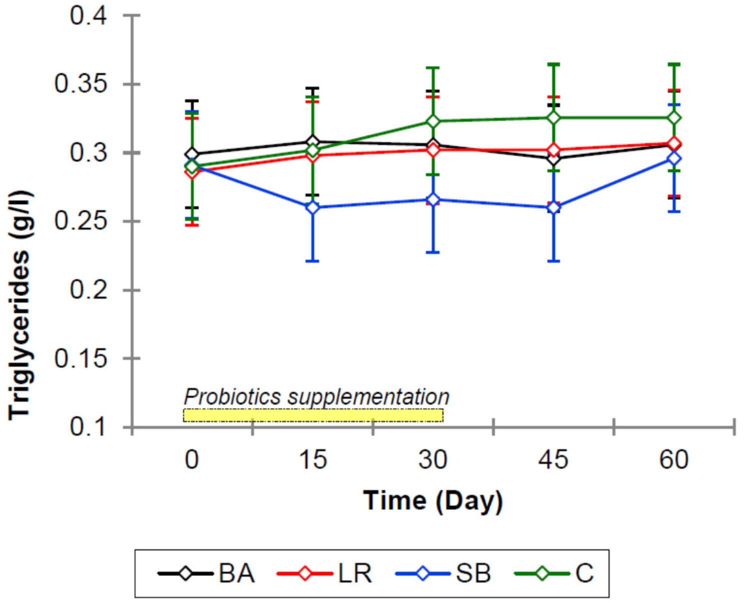

Figure S3. Effects of dietary supplementation with probiotics on blood triglycerides level of healthy rabbits. (C): control group, (BA): *Bifidobacterium animalis* subsp. *Lactis* BB-12 group, (LR*): Lactobacillus rhamnosus* GG group, (SB): *Saccharomyces boulardii* CNCM I-745 group.

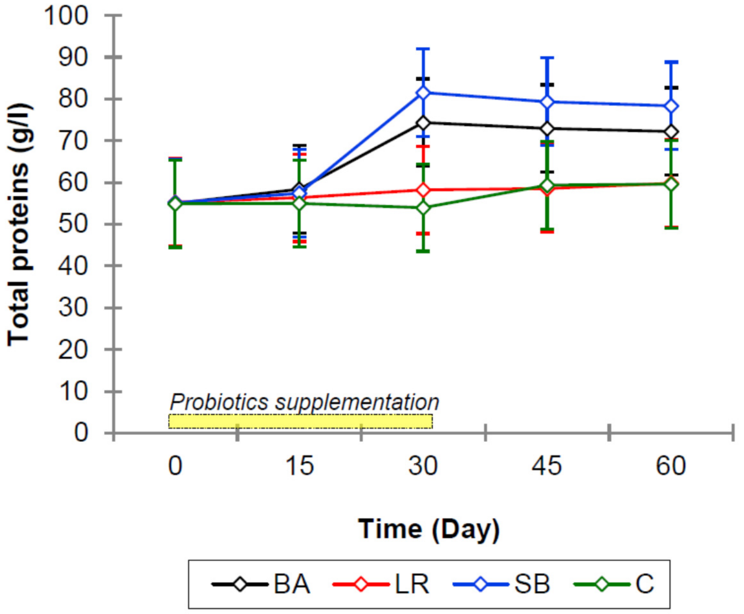

Figure S4. Effects of dietary supplementation with probiotics on blood total proteins level of healthy rabbits. (C): control group, (BA): *Bifidobacterium animalis* subsp. *Lactis* BB-12 group, (LR*): Lactobacillus rhamnosus* GG group, (SB): *Saccharomyces boulardii* CNCM I-745 group.

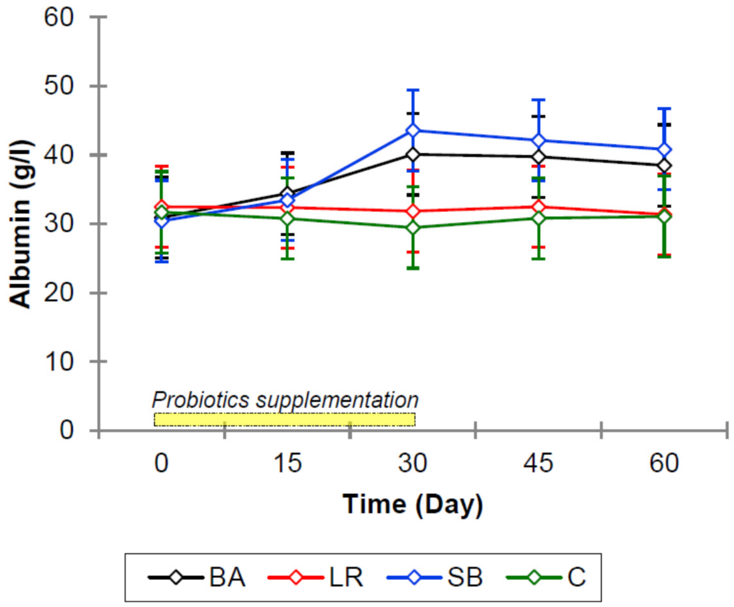

Figure S5. Effects of dietary supplementation with probiotics on blood albumin level of healthy rabbits. (C): control group, (BA): *Bifidobacterium animalis* subsp. *Lactis* BB-12 group, (LR): *Lactobacillus rhamnosus* GG group, (SB): *Saccharomyces boulardii* CNCM I-745 group.

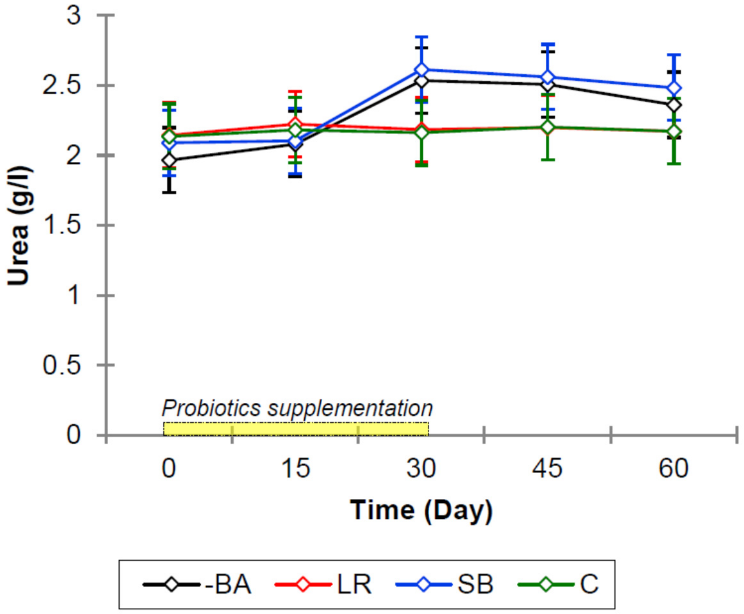

Figure S6. Effects of dietary supplementation with probiotics on blood urea level of healthy rabbits. (C): control group, (BA): *Bifidobacterium animalis* subsp. *Lactis* BB-12 group, (LR): *Lactobacillus rhamnosus* GG group, (SB): *Saccharomyces boulardii* CNCM I-745 group.

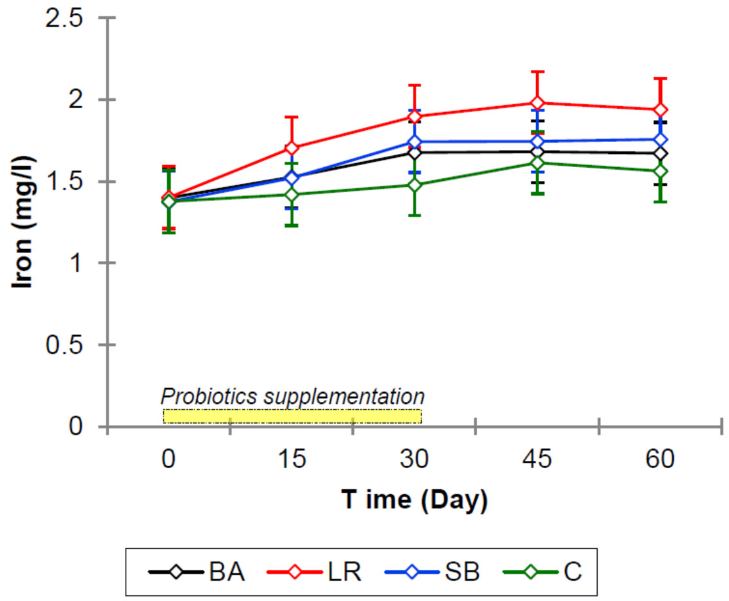

Figure S7. Effects of dietary supplementation with probiotics on blood iron level of healthy rabbits. (C): control group, (BA): *Bifidobacterium animalis* subsp. *Lactis* BB-12 group, (LR*): Lactobacillus rhamnosus* GG group, (SB): *Saccharomyces boulardii* CNCM I-745 group.

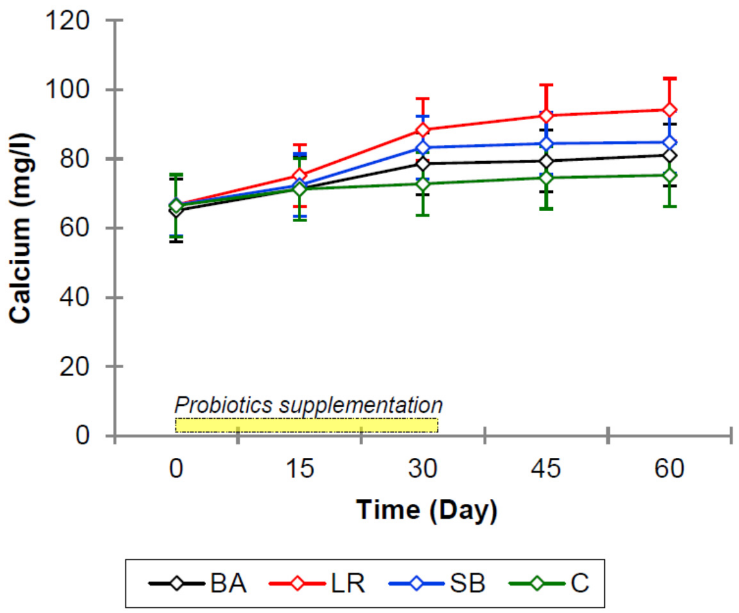

Figure S8. Effects of dietary supplementation with probiotics on blood calcium level of healthy rabbits. (C): control group, (BA): *Bifidobacterium animalis* subsp. *Lactis* BB-12 group, (LR*): Lactobacillus rhamnosus* GG group, (SB): *Saccharomyces boulardii* CNCM I-745 group.

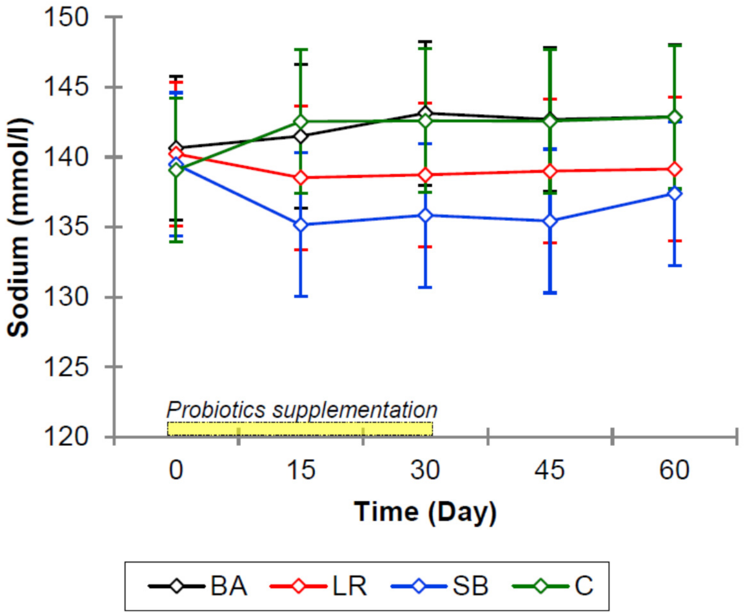

Figure S9. Effects of dietary supplementation with probiotics on blood sodium level of healthy rabbits. (C): control group, (BA): *Bifidobacterium animalis* subsp. *Lactis* BB-12 group, (LR*): Lactobacillus rhamnosus* GG group, (SB): *Saccharomyces boulardii* CNCM I-745 group.

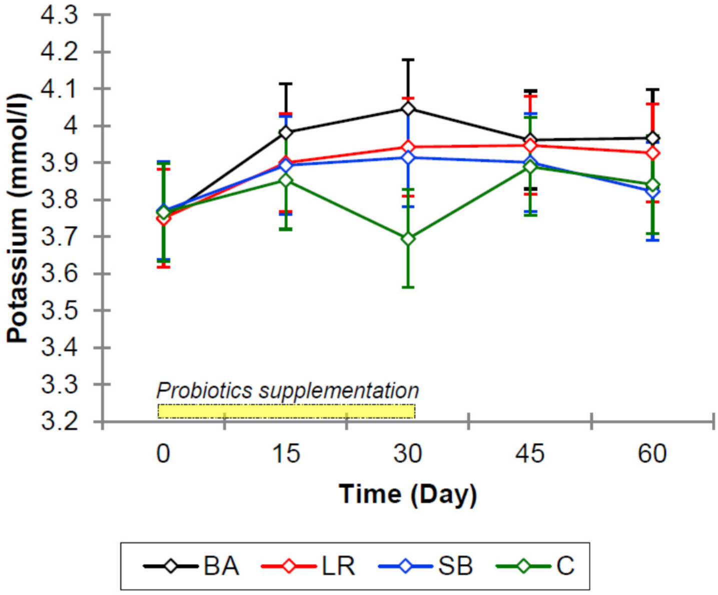

Figure S10. Effects of dietary supplementation with probiotics on blood potassium level of healthy rabbits. (C): control group, (BA): *Bifidobacterium animalis* subsp. *Lactis* BB-12 group, (LR*): Lactobacillus rhamnosus* GG group, (SB): *Saccharomyces boulardii* CNCM I-745 group.

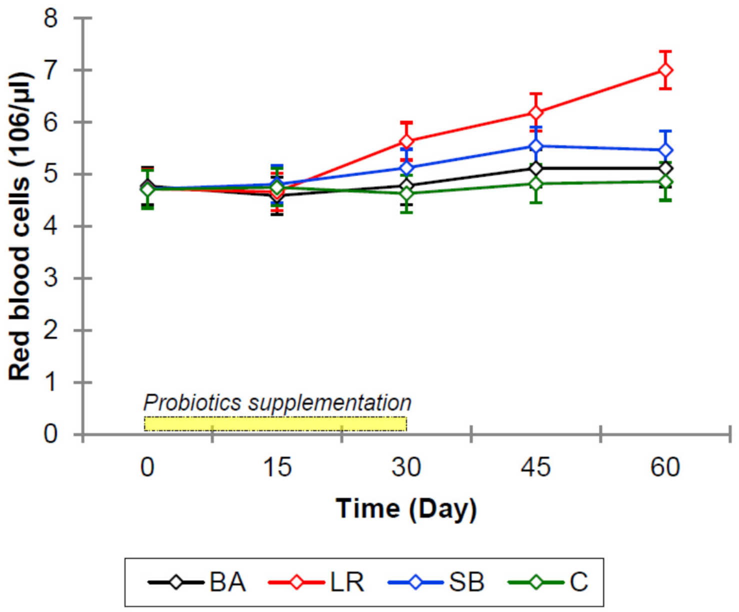

Figure S11. Effects of dietary supplementation with probiotics on number of red blood cells of healthy rabbits. (C): control group, (BA): *Bifidobacterium animalis* subsp. *Lactis* BB-12 group, (LR*): Lactobacillus rhamnosus* GG group, (SB): *Saccharomyces boulardii* CNCM I-745 group.

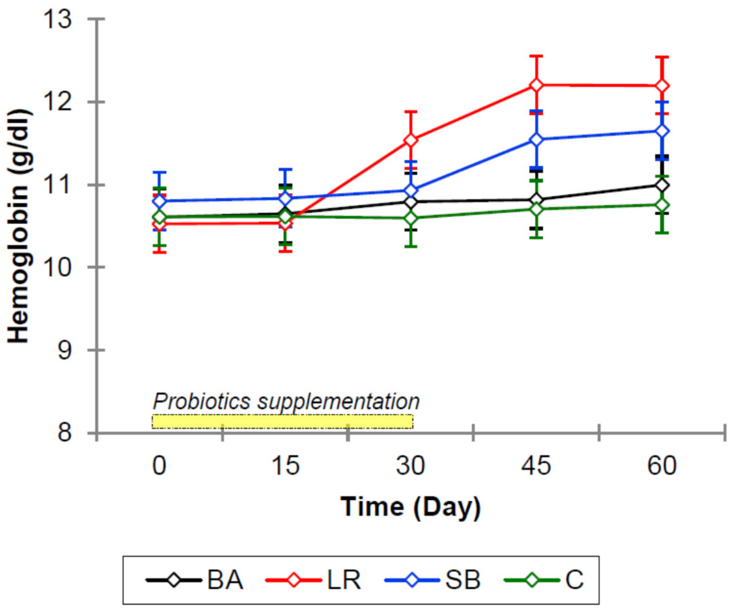

Figure S12. Effects of dietary supplementation with probiotics on number of blood hemoglobin level of healthy rabbits. (C): control group, (BA): *Bifidobacterium animalis* subsp. *Lactis* BB-12 group, (LR*): Lactobacillus rhamnosus* GG group, (SB): *Saccharomyces boulardii* CNCM I-745 group.

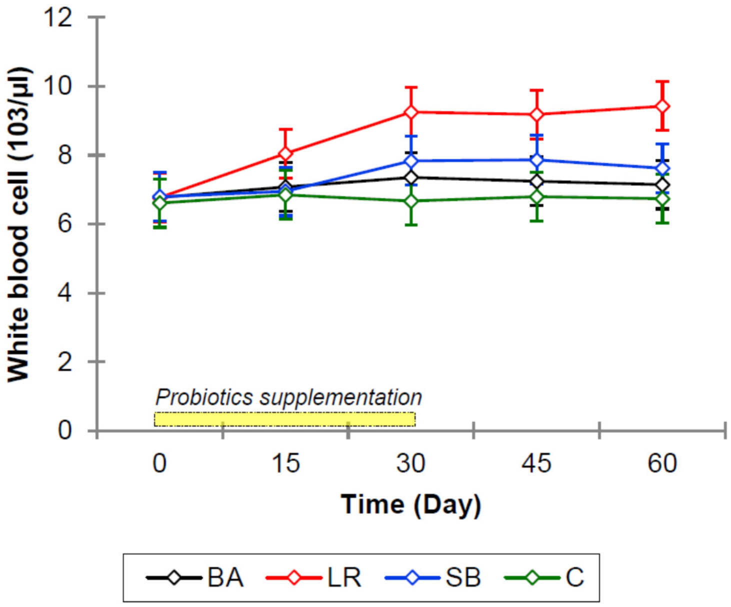

Figure S13. Effects of dietary supplementation with probiotics on number of white blood cells of healthy rabbits. (C): control group, (BA): *Bifidobacterium animalis* subsp. *Lactis* BB-12 group, (LR*): Lactobacillus rhamnosus* GG group, (SB): *Saccharomyces boulardii* CNCM I-745 group.

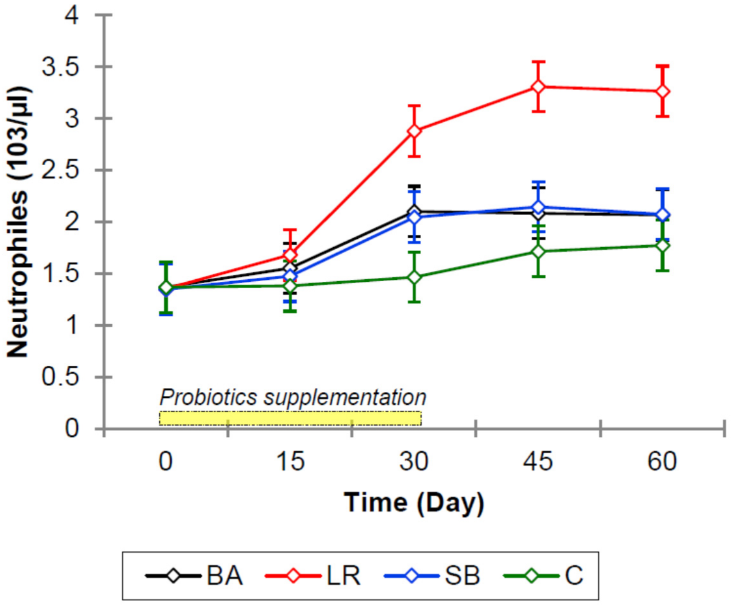

Figure S14. Effects of dietary supplementation with probiotics on number of neutrophiles of healthy rabbits. (C): control group, (BA): *Bifidobacterium animalis* subsp. *Lactis* BB-12 group, (LR*): Lactobacillus rhamnosus* GG group, (SB): *Saccharomyces boulardii* CNCM I-745 group.
